# DcR3 induces epithelial-mesenchymal transition through activation of the TGF-β3/SMAD signaling pathway in CRC

**DOI:** 10.18632/oncotarget.12639

**Published:** 2016-10-13

**Authors:** Yan-Ping Liu, Hui-Fang Zhu, Ding-li Liu, Zhi-Yan Hu, Sheng-Nan Li, He-Ping Kan, Xiao-Yan Wang, Zu-Guo Li

**Affiliations:** ^1^ Department of Pathology, Nanfang Hospital, Southern Medical University, Guangzhou, China; ^2^ Guangdong Provincial Key Laboratory of Molecular Tumour Pathology, Guangzhou, China; ^3^ Department of Pathology, School of Basic Medical Sciences, Southern Medical University, Guangzhou, China; ^4^ State Key Laboratory of Organ Failure Research, Guangdong Provincial Key Laboratory of Viral Hepatitis Research, Department of Infectious Diseases, Nanfang Hospital, Southern Medical University, Guangzhou, China; ^5^ Department of Hepatobiliary Surgery, Nanfang Hospital, Southern Medical University, Guangzhou, China

**Keywords:** decoy receptor 3, colorectal cancer, TGF-β3/SMAD signaling, metastasis, epithelial-mesenchymal transition

## Abstract

Decoy receptor 3 (DcR3), a novel member of the tumor necrosis factor receptor (TNFR) family, was recently reported to be associated with tumorigenesis and metastasis. However, the role of DcR3 in human colorectal cancer (CRC) has not been fully elucidated. In this study, we found that DcR3 expression was significantly higher in human colorectal cancer tissues than in paired normal tissues, and that DcR3 expression was strongly correlated with tumor invasion, lymph node metastases and poor prognoses. Moreover, DcR3 overexpression significantly enhanced CRC cell proliferation and migration *in vitro* and tumorigenesis *in vivo*. Conversely, DcR3 knockdown significantly repressed CRC cell proliferation and migration *in vitro*, and DcR3 deficiency also attenuated CRC tumorigenesis and metastasis *in vivo*. Functionally, DcR3 was essential for TGF-β3/SMAD-mediated epithelial-mesenchymal transition (EMT) of CRC cells. Importantly, cooperation between DcR3 and TGF-β3/SMAD-EMT signaling-related protein expression was correlated with survival and survival time in CRC patients. In conclusion, our results demonstrate that DcR3 may be a prognostic biomarker for CRC and that this receptor facilitates CRC development and metastasis by participating in TGF-β3/SMAD-mediated EMT of CRC cells.

## INTRODUCTION

Colorectal cancer (CRC) is the third most common cancer worldwide and remains a leading cause of cancer-related deaths. Although surgical techniques and adjuvant therapy have improved, the overall survival of patients with CRC has not improved dramatically [1–4]. Therefore, there is an acute need to identify new biomarkers that are capable of distinguishing between patients with poor and good prognoses.

Decoy receptor 3 (DcR3), also known as TNFRSF6B or M68, is a soluble receptor belonging to the tumor necrosis factor receptor (TNFR) superfamily [5]. Dysregulated DcR3 expression plays an important role in tumorigenesis, metastasis and immune suppression in pancreatic head cancer [6], breast cancer [7], bladder urothelial carcinoma [8], nasopharyngeal carcinoma [9], renal carcinoma [10], glioma [11] and hepatocellular carcinoma [12, 13]. However, the contribution of DcR3 to CRC and the molecular basis for this contribution have not been fully elucidated.

In this study, we found that both transcriptional expression and post-transcriptional DcR3 expression DcR3 expression were significantly up-regulated in CRC tissues. Moreover, the expression of DcR3 was significantly correlated with the clinicopathologic characteristics and overall survival of CRC patients. By manipulating DcR3 expression in CRC cells, DcR3 was found to be playing a critical role in CRC proliferation and migration *in vitro* and in tumorigenesis and metastasis *in vivo*. Interestingly, DcR3 mediated TGF-β3/SMAD-induced epithelial-mesenchymal transition (EMT) in CRC cells. Importantly, cooperation between DcR3 and TGF-β3/SMAD-EMT signaling-related protein expression was correlated with overall survival and survival time in CRC patients. Our study has identified a novel mechanism by which DcR3 regulates CRC tumorigenesis and metastasis.

## RESULTS

### DcR3 expression was associated with human CRC development, metastasis and patient survival

**Figure 1:**
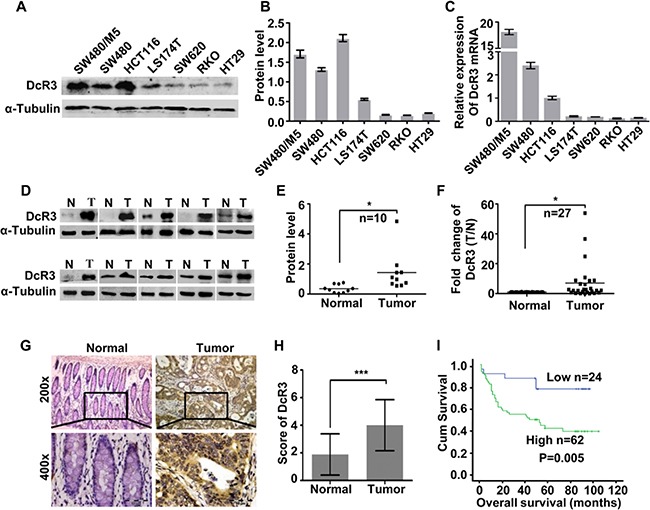
DcR3 was up-regulated in CRC tissues and positively correlated with survival in CRC patients **A.** DcR3 protein expression in CRC cell lines was detected by western blotting. **B.** Quantification of protein expression shown in A normalized to α-Tubulin. **C.** DcR3 mRNA expression in CRC cell lines was detected by qRT-PCR. **D.** DcR3 protein expression was detected in ten representative human CRC tissues. DcR3 protein expression was significantly higher in CRC tumor tissues (T) than in adjacent normal tissues (N), as detected by western blotting. **E.** Quantification of protein expression shown in D normalized to α-Tubulin. **F.** Quantitative analysis of DcR3 mRNA expression in 27 paired human CRC tissues. DcR3 mRNA expression was quantified by qRT-PCR and normalized to matched adjacent normal tissues. **G-H.** DcR3 expression in 86 paired paraffin-embedded CRC tumor tissues was detected by immunohistochemical staining. Representative DcR3 immunohistochemical staining photographs of normal tissue (normal) and tumor tissue samples, as indicated. DcR3 expression levels were significantly higher in CRC tissues than in adjacent normal tissues. Magnification: 200x, 400x. (I) DcR3 expression was positively correlated with patient survival times, as shown by Kaplan–Meier analysis, P=0.005.

At the cellular level, the expression of DcR3 protein and mRNA was detected in seven different CRC cells. Our results revealed that SW480/M5, SW480 and HCT116 cells exhibited much higher DcR3 expression levels than other cells (Figure 1A-1C), whereas that RKO and HT29 cells exhibited much lower DcR3 expression levels than other cells (Figure 1A-1C). At the tissue level, the expression of DcR3 protein was significantly up-regulated in ten CRC tissue (T) compared with their paired adjacent normal intestine epithelial tissue (N) (Figure 1D-1E). In addition, real-time PCR analysis was utilized to test the expression of DcR3 mRNA in 27 paired human CRC tissue samples. The results showed that DcR3 mRNA expression was higher in CRC tumor tissues than in normal tissues in 19 of the 27 matched tissue samples (Figure 1F).

IHC was used to detect the expression level of DcR3 in 86 cases paraffin-embedded CRC tissue samples. The results showed that the expression of DcR3 increased markedly in 62/86 CRC tissues (Figure 1G-1H). Moreover, analysis of the clinicopathologic characteristics of all 86 tissue samples showed that DcR3 levels were closely associated with tumor differentiation (P=0.006), depth of tumor cell infiltration (P=0.046) and lymphnode metastasis (P=0.012) (Supplementary Table S1). Furthermore, Kaplan–Meier survival analysis showed that patients with higher DcR3 expression levels had shorter survival times than those with lower expression levels (P=0.005) (Figure 1I). Cox regression analysis revealed that DcR3 expression, lymph node metastasis, age and infiltration were recognized as independent prognostic factors in this study (Supplementary Table S2). These data suggested that DcR3 played an important role in CRC invasion and metastasis, as well as in patient survival.

### DcR3 promoted human CRC cell proliferation and migration *in vitro*

**Figure 2:**
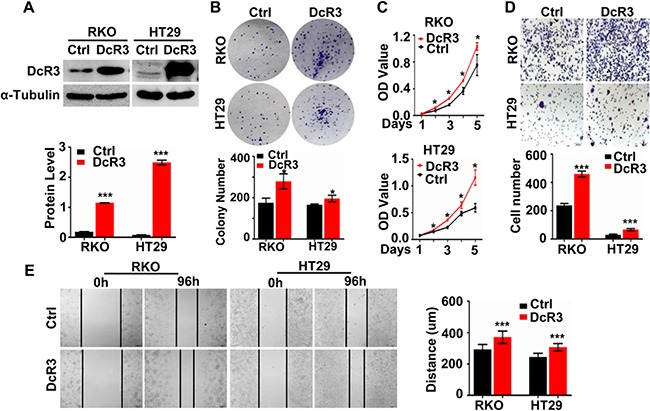
DcR3 overexpression promoted CRC cell proliferation and migration in vitro **A.** RKO and HT29 cells were stably transfected with vector (Control) or DcR3 plasmids (DcR3). DcR3 plasmids effectively increased DcR3 expression in RKO and HT29 cells (Upper panel). DcR3 protein levels were normalized to α-Tubulin (Lower panel). ***P<0.001compared to control group, n=3. **B-C.** DcR3 overexpression promoted cell proliferation, as determined by the colony formation assay (B) and the CCK8 assay (C), *P<0.05 compared to control group at each corresponding time point; n=3. (D-E) DcR3 overexpression markedly increased RKO and HT29 cell migration, as determined by the transwell migration assay **D.** and the wound-healing assay **E.** ***P<0.001 compared to control group; n=3.

To determine the role of DcR3 in CRC tumorigenesis and progression, stable DcR3 expressed cell lines RKO/DcR3 and HT29/DcR3 were made (Figure 2A). The colony formation assays and CCK8 proliferation assays results showed that DcR3 overexpression significantly promoted the proliferation of RKO and HT29 cells compared with control cells (Figure 2B-2C). Since DcR3 expression was associated with the depth of tumor infiltration and metastasis (Supplementary Table S1), we investigated whether DcR3 affects the migration of RKO and HT29 cells. Transwell migration assays and scratch wound-healing assays were carried out. As shown in Figure 2D-2E, DcR3 overexpression did promote the migration of RKO and HT29 cells.

To further confirm the impact of DcR3 expression on CRC cell proliferation and migration *in vitro*, we performed lentiviral vector-mediated shRNA knockdown of DcR3 in SW480/M5 and HCT116 cells (Supplementary Figure S1A) and carried out colony formation assays and CCK8 proliferation assays to detect CRC cell proliferation. The results revealed that DcR3 knockdown significantly inhibited colony formation and CRC growth in both SW480/M5 and HCT116 cells (Supplementary Figure S1B-S1C). Moreover, DcR3 knockdown significantly inhibited CRC cell migration (Supplementary Figure S1D-S1E). These data indicated that DcR3 facilitated CRC cell growth and migration *in vitro*.

### DcR3 promoted CRC tumor growth and metastasis *in vivo*

**Figure 3:**
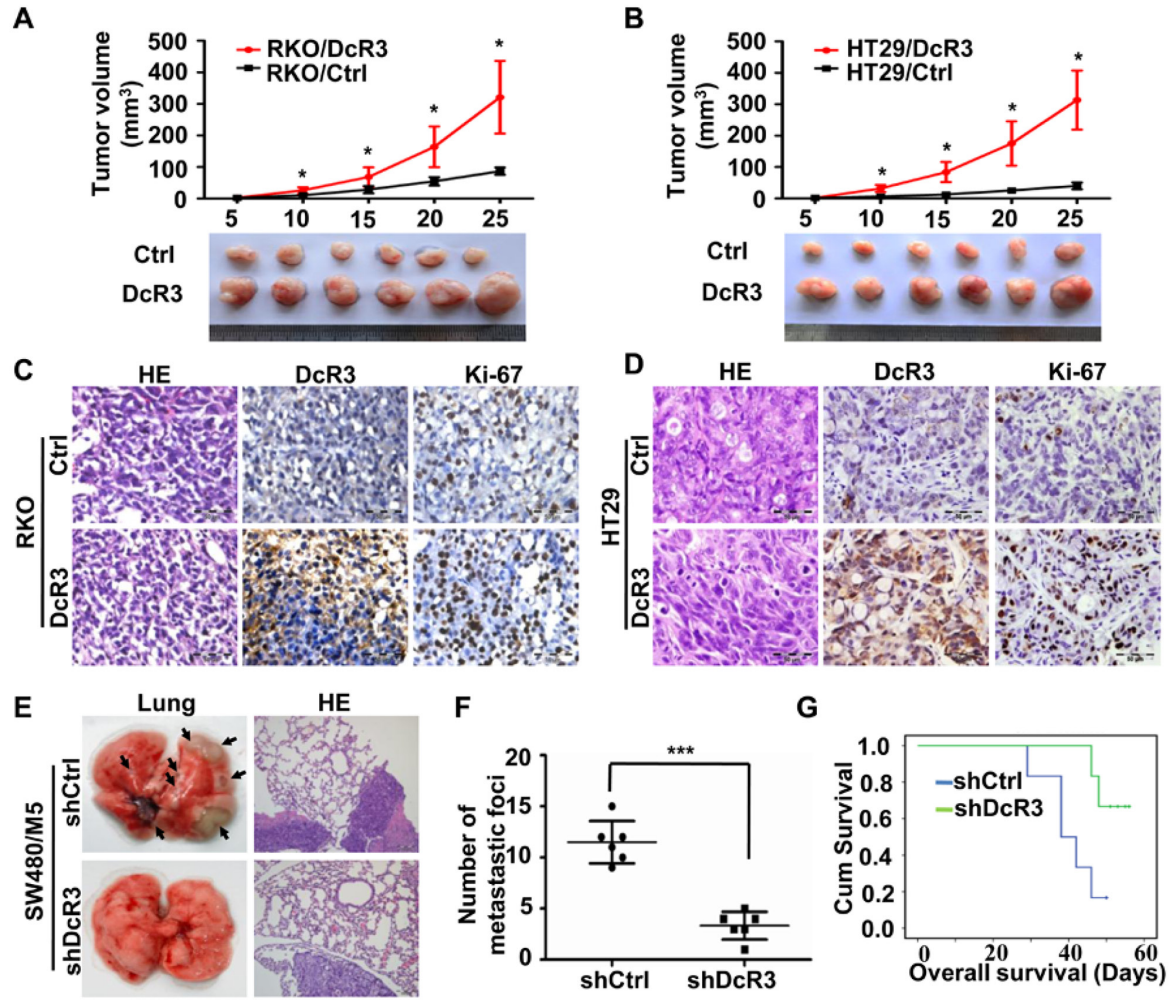
DcR3 promoted tumor growth and metastasis *in vivo* **A-B.** GFP-labeled RKO and HT29 cells stably transfected with vector (Ctrl) or DcR3 plasmids (DcR3) were injected subcutaneously into nude mice, as described in the Methods. Twenty-five days later, tumors were removed and imaged (Lower panels). Tumor growth curves were obtained using a whole-body GFP imaging system during tumor growth (Upper panels). Tumors derived from cells expressing DcR3 grew significantly larger than tumors derived from control cells. *P<0.001 compared to control group at each time point for both A and B; n=6. **C-D.** Representative photographs of H&E, DcR3 and Ki-67 immunohistochemistry staining of the primary tumor tissues from nude mice. **E.** GFP-labeled SW480/M5 cells stably transfected with control (shCtrl) or DcR3 shRNA (shDcR3) were injected into the tail veins of nude mice, as described in the Methods. Left: whole-lung images with metastatic foci, as indicated by the arrows. Right: H&E staining of lung sections showing metastatic tumors. **F.** Quantification of the numbers of metastatic foci in each mouse. More metastatic foci were detected in cells containing shCtrl than in cells containing shDcR3, ***P<0.001; n=6. **G.** DcR3 expression was positively correlated with survival time of nude mice, as shown by log-rank analysis; P=0.027.

To assess the effect of DcR3 expression on tumor growth *in vivo*, RKO/DcR3, HT29/DcR3, SW480/M5/shDcR3, HCT116/shDcR3 or control cells were injected subcutaneously into nude mice. As shown in Figure 3A-3B and Supplementary Figure S2A-S2B, xenograft tumors developed at the injection site after 5 days. During a growth period of 25 days, primary tumors derived from DcR3-overexpressing cells grew significantly faster than those derived from control cells (Figure 3A-3B (upper panels)), and primary tumors derived from DcR3-deficient cells grew at a significantly slower rate than those derived from control cells (Supplementary Figure S2A-S2B (upper panels)). Moreover, tumors derived from DcR3-overexpressing cells were significantly larger than those derived from control cells (Figure 3A-3B (lower panels)), and tumors derived from DcR3-deficient cells were significantly smaller than those derived from control cells (Supplementary Figure S2A-S2B (lower panels)).

IHC staining confirmed that tumors derived from CRC cells stably expressing DcR3 plasmids exhibited higher DcR3 expression levels and higher cell proliferation indices, as shown by Ki-67 staining, than tumors derived from control cells (Figure 3C-3D). While knockdown of DcR3 expression in SW480/M5 and HCT116 showed the opposite results (Supplementary Figure S2C-S2D). These data indicate that DcR3 facilitates CRC cell growth *in vivo*.

To determine the effect of DcR3 expression on CRC metastasis *in vivo*, we established a tail vein xenograft model to investigate CRC cell lung metastasis. SW480/M5 cells stably expressing control or DcR3 shRNA were injected into 6 mice per group. We found that the SW480/M5/shCtrl group exhibited more metastatic foci than the SW480/M5/shDcR3 group (Figure 3E-3F). Moreover, DcR3 knockdown prolonged the survival time of nude mice (Figure 3G). These results indicate that DcR3 promotes CRC cell metastasis *in vivo*.

### DcR3 facilitated epithelial-mesenchymal transition (EMT) of CRC cells

**Figure 4:**
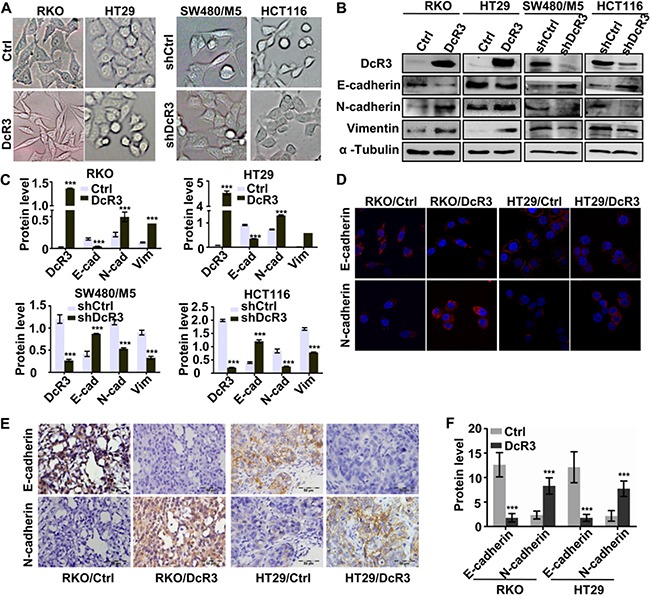
DcR3 facilitated CRC epithelial-mesenchymal transition **A.** DcR3 overexpression in RKO and HT29 cells caused morphological alteration sresembling EMT, while konckdown of DcR3 in SW480/M5 and HCT116 caused and reverse result. **B.** The expression of mesenchymal and epithelial markers in RKO and HT29 cells transfected with vector (control) or DcR3 plasmid (DcR3) and SW480/M5 and HCT116 cells transfected of control (shCtrl) or DcR3 shRNA (shDcR3) was detected by western blotting. **C.** Quantification of protein expression shown in B normalized to α-Tubulin. *P<0.05 compared to control group; n=3. **D.** Immunostaining of mesenchymal and epithelial markersin RKO and HT29 cells transfected with vector (control) or DcR3 plasmid (DcR3), as indicated. Magnification: 1800x. **E.** EMT-related protein immunohistochemical staining of primary tumor tissues derived from control or DcR3-containing RKO or HT29 cells, as indicated. Magnification: 200x. **F.** Quantification of protein expression was assessed by measuring IHC staining intensity. ***P<0.001 compared to control group for each corresponding protein; n=6. DcR3 overexpression in RKO and HT29 increased N-cadherin and vimentin expression and decreased E-cadherin expression.

Since DcR3 overexpression altered RKO and HT29 cell morphology such that both cell lines exhibited a spindle-like, fibroblastic cell morphology (Figure 4A), and high DcR3 expression levels were correlated with CRC metastasis in humans (Supplementary Table S1), we hypothesized that DcR3 participates in the epithelial-mesenchymal transition (EMT) of CRC cells, which is a key event associated with tumor invasion and metastasis [14–16]. We found that DcR3 overexpression in RKO and HT29 cells significantly increased the expression of N-cadherin and vimentin, and decreased the expression of E-cadherin (Figure 4B-4D), while knockdown of DcR3 in SW480/M5 and HCT116 showed reverse result. IHC staining confirmed that tumors derived from CRC cells stably expressing DcR3 plasmids exhibited higher levels of N-cadherin expression and lower levels of E-cadherin expression than tumors derived from control cells (Figure 4E-4F). These results indicated that DcR3 facilitated EMT of RKO and HT29 cells.

### DcR3 was required for TGF-β3/SMAD signaling-induced EMT

**Figure 5:**
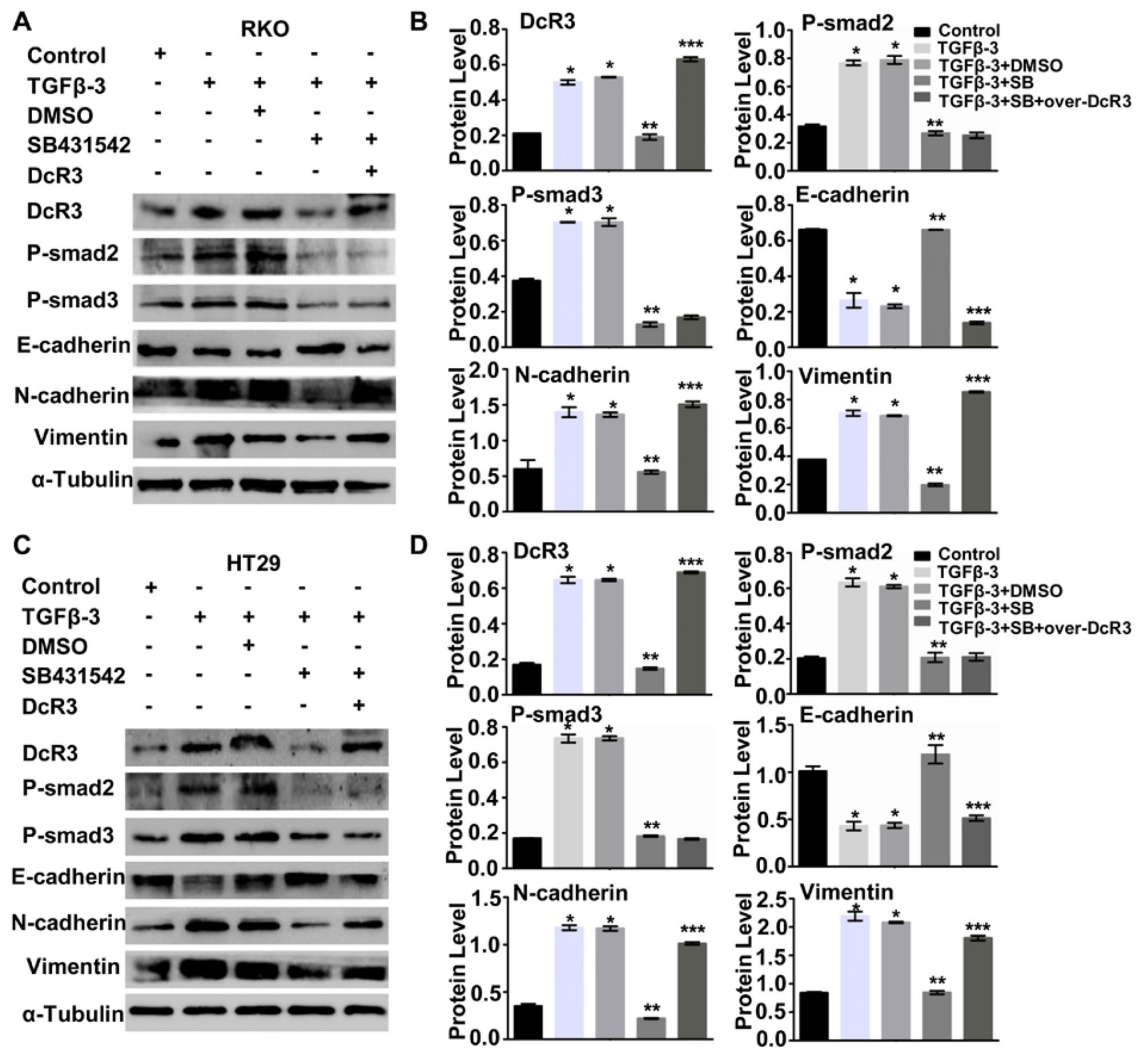
DcR3 was required for TGF-β3/SMAD signaling-induced EMT **A.** TGF-β3 promoted smad signaling and DcR3 expression and induced RKO cell EMT. SB431542 (SB) attenuated P-Smad2, P-smad3 and DcR3 expression and inhibited RKO cell EMT. DcR3 overexpression in P-smad2- and P-Smad3-deficient cells rescued TGF-β3-induced EMT. **B.** Quantification of protein expression shown in (A) normalized to α-Tubulin.*P<0.001 compared to untreated group (control); **P<0.001 compared to DMSO-treated group (TGF-β3+DMSO); ***P<0.001 compared to SB431542 (SB)-treated group (TGF-β3 + SB431542) for each protein; n=3. **C.** TGF-β3 promoted smad signaling and DcR3 expression and induced EMT of HT29 cells. SB431542 (SB) attenuated P-Smad2, P-smad3 and DcR3 expression and inhibited EMT of HT29 cells. DcR3 overexpression in P-smad2- and P-Smad3-deficient cells rescued TGF-β3-induced EMT. **D.** Quantification of protein levels shown in (C) normalized to α-Tubulin. *P<0.001 compared to untreated groups (control); **P<0.001 compared to DMSO-treated group (TGF-β3+DMSO); ***P<0.001 compared to SB431542 (SB)-treated groups (TGF-β3+SB431542) for each protein; n=3.

Finally, we explored the possible mechanism of DcR3 accelerating proliferation and invasion of CRC cells. Several reports have shown that TGF-β3 regulates epithelial-mesenchymal transition in cancer [17–20]. Thus, we investigated whether TGF-β3 induces EMT of RKO and HT29 cells. Our results revealed that TGF-β3 induced EMT of RKO and HT29 cells, as shown by the decreased expresson of E-cadherin and increased expression of N-cadherin and vimentin. Importantly, TGF-β3 also enhanced smad2/3 phosphorylation in RKO and HT29 cells. Interestingly, TGF-β3 enhanced DcR3 expression in RKO and HT29 cells (Figure 5A-5D).

To determine whether the SMAD signaling pathway is required for TGF-β3-induced EMT, we used SB431542 (SB) to inhibit smad2/3 phosphorylation in RKO and HT29 cells treated with TGF-β3. As shown in Figure 5A-5D, inhibition of smad2/3 phosphorylation restored E-cadherin expression and attenuated N-cadherin and vimentin expression in both RKO and HT29 cells. These results suggested that activated smad2/3 signaling is required for TGF-β3-induced EMT of RKO and HT29 cells.

Notably, inhibition of smad2/3 phosphorylation by SB431542 (SB) also blocked TGF-β3-induced DcR3 protein expression (Figure 5A-5D). We therefore hypothesized that DcR3 is required for TGF-β3/SMAD signaling-induced EMT and thatDcR3 overexpression can rescue TGF-β3/SMAD signaling-induced EMT. DcR3 overexpression attenuated E-cadherin expression, but N-cadherin and vimentin expression was altered owing to the inhibition of smad2/3phosphorylation in TGF-β3-treated cells (Figure 5A-5D). These results clearly indicate that DcR3 mediates TGF-β3/SMAD-induced EMT of CRC cells.

### DcR3 was positively correlated with EMT and TGF-β3/SMAD signaling in CRC tissues

**Figure 6:**
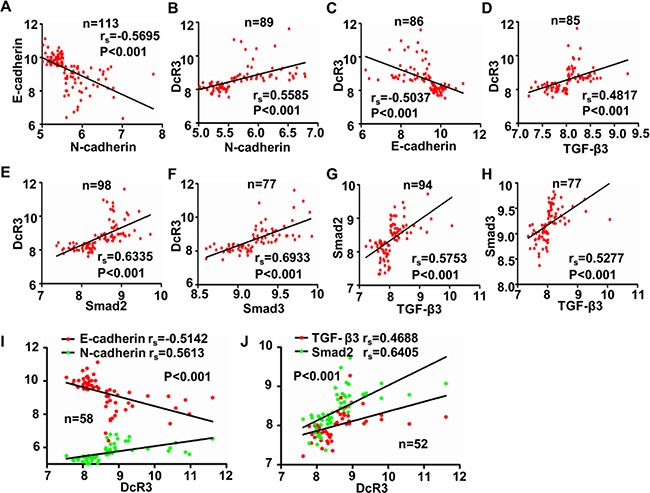
DcR3 was positively correlated with EMT and TGF-β3/SMAD pathway activity in CRC tissues **A-J.** Analysis of the correlations between N-cadherin and E-cadherin expression (A, r_s_=−0.5695, P<0.001), DcR3 and N-cadherin expression(B, r_s_=−0.5585, P<0.001), DcR3 and E-cadherin expression (C, r_s_=−0.5037, P<0.001), DcR3 and TGF-β3 expression (D, r_s_=0.4817, P<0.001), DcR3 and smad2 expression(E, r_s_=0.6335, P<0.001), DcR3 and smad3 expression(F, r_s_=0.6933, P<0.001), TGF-β3 and smad2 expression(G, r_s_=−0.5753, P<0.001), and TGF-β3 and smad3 expression(H, r_s_=−0.5277, P<0.001). High DcR3 expression was positively correlated with elevated expression of N-cadherin, TGF-β3, smad2, and smad3, and decreased expression of E-cadherin in human CRC tissues.

To analyze the correlation between DcR3 expression and TGF-β3/SMAD signaling in CRC tissues, we analyzed the association between DcR3 expression and the TGF-β3/SMAD pathway in a public clinical microarray dataset of 177 CRC tissues [21]. We found that DcR3 expression was positively correlated with N-cadherin expression and negatively correlated with E-cadherin expression, suggesting that DcR3 is associated with the activation of EMT in CRC (Figure 6A-6C). Importantly, DcR3 was positively correlated with TGF-β3, smad2 and smad3 expression in CRC tissues (Figure 6D-6F). TGF-β3 was also positively correlated with both smad2 and smad3 expression (Figure 6G-6H), indicating that DcR3 mediates TGF-β3/SMAD signaling. Taken together, these data indicated that DcR3 is positively correlated with EMT and TGF-β3/SMAD signaling in CRC tissues.

### Cooperation between DcR3 and TGF-β3/SMAD-EMT signaling-related protein expression was correlated with survival and survival time in CRC patients

**Figure 7:**
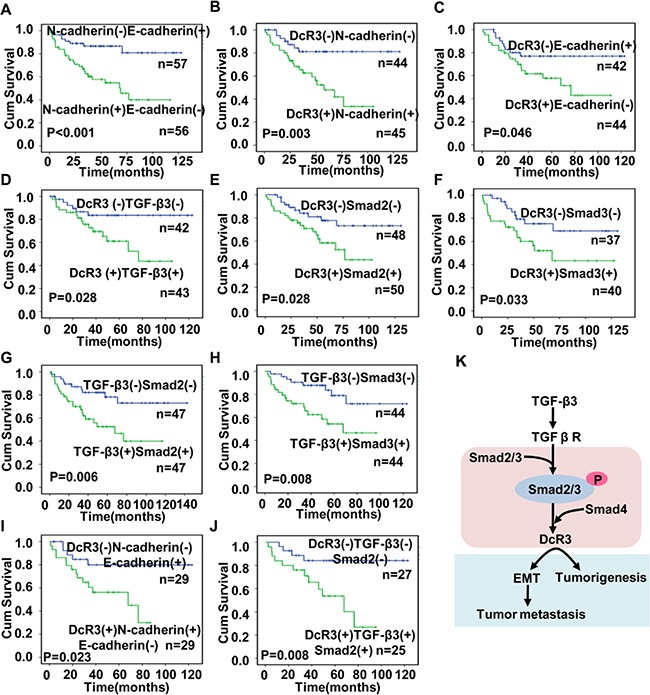
Cooperation between DcR3 and TGF-β3/SMAD-EMT signaling-related protein expression correlated with survival and survival times in CRC patients **A-J.** Kaplan-Meier analysis of the relationship ofconcurrent N-cadherin and E-cadherin expression (A, P<0.001), DcR3 and N-cadherin expression (B, P=0.003), DcR3 and E-cadherin expression (C, P=0.046), DcR3 and TGF-β3 (D, P=0.028), DcR3 and smad2 expression (E, P=0.028), DcR3 and smad3 expression (F, P=0.033), TGF-β3 and smad2 expression (G, P=0.006), TGF-β3 and smad3 expression (H, P=0.008), DcR3/N-cadherin and E-cadherinexpression (I, P=0.023), and DcR3/TGF-β3 and smad2expression (J, P=0.008) with recurrence and overall survival in CRC patients. Activation of the TGF-β3/SMAD pathway was associated with shorter overall survival times. Patients expressing DcR3 and exhibiting TGF-β3/SMAD pathway activity had poorer prognoses. (K) Summary diagram showing cooperation between DcR3 and TGF-β3/SMAD-EMT signaling-related protein expression in CRC.

In addition, we performed Kaplan–Meier survival analyses and found that E-cadherin**^(−)^** and N-cadherin^(+)^, DcR3^(+)^ and N-cadherin^(+)^ and DcR3^(+)^ and E-cadherin**^(−)^** patients had shorter overall survival times than E-cadherin**^(+)^** and N-cadherin^(−)^, DcR3^(−)^ and N-cadherin^(−)^ and DcR3^(−)^ and E-cadherin**^(+)^** patients, suggesting that cooperation between DcR3 and EMT-related protein expression was strongly correlated with survival and survival time of patients (Figure 7A-7C). Moreover, DcR3**^(+)^** patients exhibiting activated TGF-β3/SMAD signaling-related protein expression had a poorer prognosis (Figure 7D-7F) than DcR3**^(−)^** patients exhibiting inactivated TGF-β3/SMAD signaling-related protein expression, and patients with an activated TGF-β3/SMAD signaling pathway exhibited shorter overall survival times (Figure 7G-7H). Interestingly, DcR3**^(+)^** patients with activated TGF-β3/SMAD-EMT signaling-related protein expression faced poorer prognoses than other CRC patients (Figure 7I-7J). These data indicated that cooperation between DcR3 and TGF-β3/SMAD-EMT signaling-related protein expression correlates with survival and survival time of CRC patients and that DcR3 promotes TGF-β3/SMAD-mediated CRC metastasis.

## DISCUSSION

As previously reported, DcR3 has been detected in embryo lung [22], brain, liver, and some autoimmune diseases [23–26]. Recently, DcR3 has attracted extensive attention because its aberrant expression has been detected in various types of malignant tumors, especially hepatocellular carcinoma [12], pancreatic carcinoma [27], nasopharyngeal carcinoma [9], ovarian cancer [28], gastric carcinoma [29] and glioblastoma [30]. DcR3 has also been linked to the occurrence, development and prognosis of these malignant tumors [23]. However, the role of DcR3 in CRC development has not been fully elucidated. In the present study, we examined the role of DcR3 in CRC development. Our findings suggested that DcR3 played an important role in CRC progression because DcR3 promoted CRC cell proliferation *in vitro* and CRC tumor growth *in vivo*. Moreover, similar to the findings of Zong, L [31], DcR3 expression in human CRC tissues was correlated with CRC differentiation, depth of tumor infiltration and lymphnode metastasis, indicating that DcR3 facilitates CRC metastasis. This finding is supported by the observation that DcR3 deficiency decreased CRC cell migration *in vitro* and attenuated CRC cell lung metastasis *in vivo*. Most importantly, DcR3 expression was associated with prognosis and survival of CRC patients. These observations indicate that DcR3 may serve as a valuable biomarker for monitoring CRC development in humans.

Tumor metastasis is facilitated by many different mechanisms. For instance, EMT is associated with cancer cell invasion and metastasis [32]. DcR3 appears to regulate CRC tumor metastasis by mediating the EMT of CRC cells, which has been confirmed as one of the mechanisms underlying CRC metastasis [33]. DcR3 overexpression significantly increased vimentin and N-cadherin expression and decreased E-cadherin expression both *in vitro* and *in vivo*—findings consistent with those of EMTs observed in cancer cells [34]. These observations indicated that DcR3 facilitates the epithelial-mesenchymal transition (EMT) of CRC as a means of promoting metastasis.

Transforming growth factor-β comprises three isoforms, TGF-β1, TGF-β2 and TGF-β3, which can regulate the growth, differentiation, and migration of nearly all cell types [35–37]. Several reports have indicated that multiple genes and proteins are associated with TGF-β/SMAD signaling-induced epithelial-mesenchymal transition, but most of these reports focused on the function of TGF-β1 in TGF-β/SMAD signaling. Only a few reports have focused on TGF-β3 [17–20]. Our data indicated that TGF-β3 not only simultaneously induced both DcR3 and phosphorylated smad2/3 expression in CRC cells but also activates EMT. Furthermore, the smad2/3 phosphorylation inhibitor SB431542 (SB) repressessmad2/3 phosphorylation and DcR3 expression and inhibits EMT of CRC cells. Thus, we hypothesized that DcR3 was required for TGF-β3/SMAD signaling-induced EMT. The role of DcR3 inTGF-β3/SMAD signaling-induced EMT was supported by several lines of evidence. First, DcR3 overexpression rescued the attenuation of TGF-β3/SMAD-induced EMT facilitated by the smad2/3 phosphorylation inhibitor SB431542 (SB). Second, analysis of the association between DcR3 and TGF-β3/SMAD-EMT signaling-related protein expression in a public clinical microarray dataset [21] indicated that DcR3 expression was positively correlated with TGF-β3/SMAD-EMT signaling in CRC tissues. Finally, cooperation between DcR3 and TGF-β3/SMAD-EMT signaling related protein expression was strongly correlated with patient survival and survival time. DcR3^(+)^ patients exhibiting activated TGF-β3/SMAD signaling-related protein expression had a poorer prognosis. Thus, DcR3 was essential for TGF-β3/SMAD-mediated CRC metastasis.

In conclusion, our study demonstrated that DcR3 enhanced tumorigenes and metastasis in patients with CRC. The function of DcR3 may be partially facilitated by activation of the TGF-β3/SMAD signaling pathway, resulting in EMT in CRC cells. Because DcR3 expression was associated with tumor differentiation, tumor infiltration depth, tumor metastasis, and lower survival, DcR3may be useful as a biomarker for making early diagnoses, predicting prognoses, and improving disease management in patients with CRC.

## MATERIALS AND METHODS

### Clinical specimens and cell culture

The use of 86 archived, formalin-fixed paraffin-embedded human colorectal carcinoma tissues for this study was approved by the ethics committee of Nanfang Hospital, Southern Medical University, China. Before using these clinical materials for research purposes, we obtained informed consent from all the patients. None of the patients in question had received any preoperative chemotherapy or radiotherapy.

CRC cell lines SW480, HCT116, LS174T, SW620, RKO and HT29 were obtained from the American Type Culture Collection (ATCC). SW480/M5 was established at our laboratory [38]. All cell lines were cultured in RMPI-1640 medium containing 10% fetal bovine serum (Gibco, USA) in 5% CO_2_ at 37 °C.

### RNA isolation and quantitative real-time PCR

Total RNA was extracted from tissues or cells using TRIzol reagent (Takara, Japan), according to the manufacturer's instructions. RNA was reverse transcribed to cDNA with a Reverse Transcription Kit (Takara). Quantitative real-time PCR (qRT-PCR) analysis was performed using SYBR Green Master Mix (Takara), and the analysis was performed in triplicate. The results were normalized to the expression of GAPDH. The DcR3 primers were as follows: (forward) 5′-GTA CGC GGA GTG GCA GAA A-3′ and (reverse) 5′-CAG AGG ACG TTG CAG TAG C-3′ (206bp). The GAPDH primers were as follows: (forward) 5′-ACA GTC AGC CGC ATC TTC TT-3′ and (reverse) 5′-GAC AAG CTT CCC GTT CTC AG-3′ (351bp).

### Western blotting

Proteins were extracted using lysis buffer and quantified by bicinchoninic acid (BCA) protein quantitative assay (KeyGen Biotech, China). Protein lysates were separated using 10% SDS-PAGE and transferred onto PVDF membranes (Roche, Switzerland). Then, the membranes were incubated with specific antibodies against DcR3 (Abcam, England), p-Smad2, p-Smad3, E-cadherin, N-cadherin and vimentin (Cell Signaling Technology, USA), followed byincubation with the appropriate second antibodies. Finally, the membranes were detected using an enhanced chemiluminescence (ECL) detection system (FDbio, China), according to the manufacturer's instructions.

### Construction of lentivirus and stable cell lines

According to the manufacturer's instructions, lentiviral constructs containing the indicated DcR3-repressing shRNA sequence (TCATCGACTTTGTGGCTTT) purchased from Gene Pharma (Suzhou, China) were used to establish cell lines constitutively repressing DcR3. Lentiviral constructs not repressing any known human genes were used to establish a control cell line. The DcR3 sequence was synthesized and subcloned into a GV358 vector (Genechem, China). Lentiviral vector encoding the human DcR3 gene was designated LV-DcR3. An empty vector was used as a control and designated LV-control. Antibiotic-resistant transfected cells were selected via 5 ug/ml puromycin (Sigma, USA) administration in the culture medium. DcR3 transfection efficiency was examined by western blotting.

### Immunohistochemistry

Following fixation and embedding, surgical specimens were cut into 5 μm-thicksections. According to the specifications of the S-P kit, tissue sections were retrieved with citrate buffer, incubated with an anti-DcR3 primary antibody (Abcam, England), and then detected with an avidin-biotin complex with 3, 3′-diaminobenzidine. The degree of staining was observed and scored independently by two pathologists. Cytoplasmic immune staining intensity was rated as follows: 0(no staining), 1(yellow or light brown, weak staining), 2 (brown, moderate staining) and 3(dark brown, strong staining). DcR3 staining quality was rated as follows: 0(no staining), 1(<30%), 2(30%-70%) and 3(>70%). Tumor tissue intensity was scored via summation as follows: 0-1 (−), 2–3 (+), 4 (++), and 5–6 (+++). Tissues scored 0-1 (−)/2-3 (+) were classified into the low-expression group, and tissues scored 4 (++)/5-6 (+++) were classified into the high-expression group [12].

### Immunofluorescence

After being fixed with 4% paraformaldehyde for 30 min and permeabilized with 0.5% Triton X-100 for 10 min at room temperature, cells seeded on confocal dishes were incubated with primary antibodies at 4 °C overnight. Then, the cells were washed with PBS and incubated with the appropriate fluorescent secondary antibody in the dark at room temperature for 1 h. Finally, the confocal dishes were mounted using an anti-fade mounting solution containing 4, 6-diamidino-2-phenylindole (DAPI) and washed with PBS. Staining was examined, and images were captured using an Olympus FV1200 confocal laser scanning microscope.

### *In vitro* cell growth assay and colony-formation assay

Cells were seeded in 96-well plates (1×10^3^/well). Cell proliferation was evaluated by Cell Counting Kit-8 (CCK-8, Japan) assay, according to the manufacturer's protocols. Briefly, 10 μl of CCK-8 solution was added to the culture medium and incubated for 2 h in 5% CO_2_ at 37 °C. Then, the absorbance at 450 nm was measured. The cell proliferation assay was performed on days 1, 2, 3, 4 and 5.

For the colony-formation assay, cells were seeded in 6-well plates (400/well). After being cultured for two weeks, the cells were washed twice with PBS, fixed with 4% paraformaldehyde, and then stained with hematoxylin for 30 minutes. Finally, the number of colonies containing more than 50 cells under was counted under a microscope. All experiments were repeated three times.

### *In vitro* scratch wound-healing assay and migration assay

Cell migration ability was assessed by the wound healing assay and the migration assay. For the wound healing assay, artificial wounds were scratched on a confluent cell monolayer using sterile tips, and wound healing images were taken at the appropriate time. For the migration assay, 1×10^5^ cells in 200 μl of serum-free medium were placed into the top chamber of a transwell chamber (BD Biosciences, USA), which featured a pore size of 8 μm. The matched lower chamber contained 500 μl of 10% FBS medium, which served as a chemoattractant. After incubating for the appropriate time, the cells were fixed with formaldehyde and stained with hematoxylin for 30 minutes. Then, the cells that migrated to the basal portion of the membrane in the lower compartment of the chamber were counted in 5 random visual fields using a light microscope (×200).

### *In vivo* tumor growth and metastasis assay

Four to six-week-old athymic BALB/c nude mice were obtained from the Experimental Animal Centre of Southern Medical University (permission number: SCXK2011-0015). All animal experiments were conducted such that the animals received ethical and humane treatment, in accordance with a license from the Guangdong Provincial Bureau of Science, and all procedures were approved by the Institutional Animal Care and Use Committee of Southern Medical University.

For the *in vivo* tumor growth assay, after being re-suspended in serum-free medium, 5×10^6^ cells were injected subcutaneously into the left or right flank of nude mice (n=6 per group). Twenty five days later, tumors were removed and measured. For the metastasis model, 2×10^6^ cells were injected into the tail veins of six-week-old athymic BALB/c nude mice (n=6 per group). Eight weeks later, the mice were sacrificed via cervical dislocation, and all organs were removed and formalin-fixed for histological analysis.

### Accession numbers for data sets

The clinical data sets reanalyzed in this study came from the GEO database (GSE17538).

### Statistical analysis

The quantitative results of all experiments are expressed as the mean ± SD. Differences among/between sample groups were analyzed by one-way ANOVA or the independent-samples t test. Relationships between DcR3 expression and clinicopathologic characteristics were tested using Pearson x^2^test. Survival curves were plotted using the Kaplan–Meier method and compared by the log-rank test using SPSS 13.0 software. Differences were considered significant if P<0.05*; P<0.01**; P<0.001***.

## SUPPLEMENTARY MATERIALS FIGURES AND TABLES


