# Supplementary material for: DcR3 induces epithelial-mesenchymal transition through activation of the TGF-β3/SMAD signaling pathway in CRC
Source: Oncotarget. 2016 Oct 13;7(47):77306–18. doi: 10.18632/oncotarget.12639 (PMC5363587; doi:10.18632/oncotarget.12639)
Supplement: Supplementary file 1 [file oncotarget-07-77306-s001.pdf]

DcR3 induces epithelial-mesenchymal transition through activation of the TGF-β3/SMAD signaling pathway in CRC

SUPPLEMETARY FIGURES AND TABLES

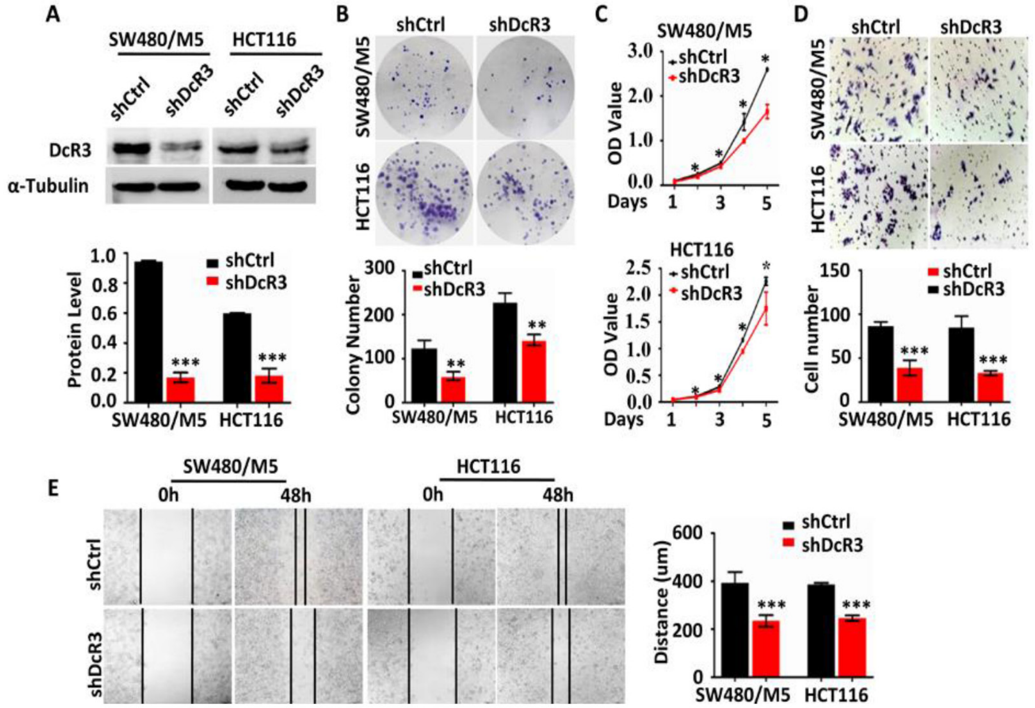

Supplemetary Figure S1: Knockdown of DcR3 expression inhibited CRC cells proliferation and migration *in vitro*. A. SW480/M5 and HCT116 cells were stably tranfected with scramble (shCtrl) or DcR3 shRNA (shDcR3), DcR3 shRNA effectively blocked its protein expression in SW480/M5 and HCT116 cells (Upper panels). DcR3 protein levels were normalized to α-Tubulin (Lower panel). ***P<0.001 compared to control group, n=3. **B-C.** Knockdown of DcR3 inhibited cell proliferation as measured by colony formation assay (B) and CCK8 assay (C), *P<0.05, **P<0.01 compared to shCtrl groups or that in each corresponding time point, n=3. **D-E.** Knockdown of DcR3 markedly attenuated SW480/M5 and HCT116 cells migration as measured by transwell migration assays (D) and wound-healling assay (E). ***P<0.001 compared to control group, n=3.

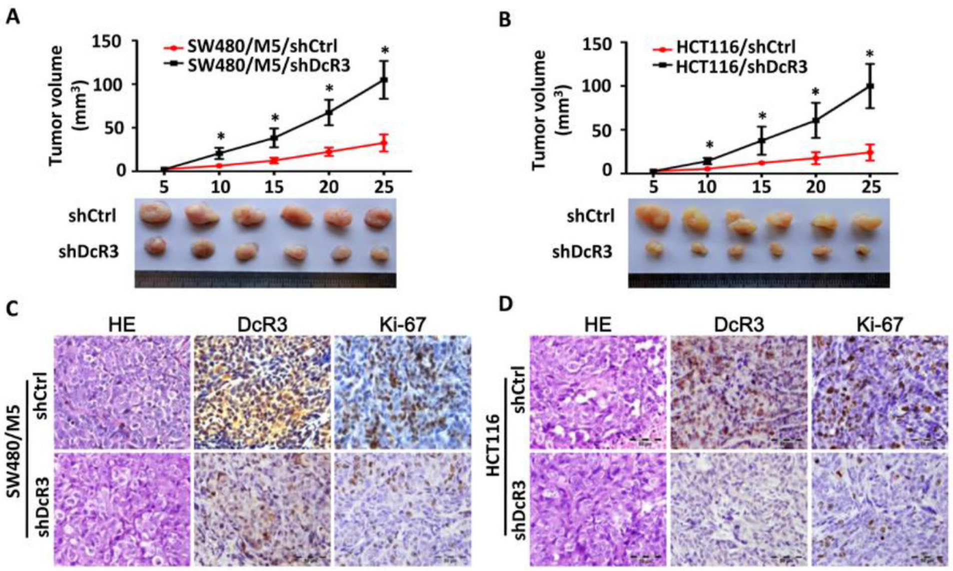

Supplemetary Figure S2: Knockdown of DcR3 inhibited tumor growth and metastasis *in vivo*. A-B. GFP-labeled SW480/M5 and HCT116 cells with stable transfection of control (shCtrl) or DcR3 shRNA (shDcR3) were injected subcutaneously into nude mice as described in Methods. 25 days later, the tumors were removed and imaged (lower panels). Tumor growth curves were obtained by using a whole-body GFP imaging system during the growth of the tumors (upper panels). Tumors derived from cells expressing shDcR3 grew significantly smaller than that from cells with shCtrl. *P<0.001 compared to shCtrl group in each corresponding time point for both A and B, n=6. **C-D.** Representative photographs of H&E, DcR3 and Ki-67 immunohistochemistry staining of the primary tumor tissues from nude mice.

Supplementary Table S1: Clinicopathological characteristics of patients and DcR3 protein expression in CRC tissues

Characteristic		DcR3 Expression: No. of Patients (%)		P
		High	Low	
Age(y)	≥50	49 (72.1)	19 (27.9)	0.989
	<50	13(72.2)	5 (27.8)	
Gender	Male	40(70.2)	17 (29.8)	0.578
	Female	22(75.9)	7 (24.1)	
Differentiation	High	16(53.3)	14 (46.7)	0.006
	Moderate	27 (79.4)	7 (20.6)	
	Low	19(86.4)	3 (13.6)	
Infiltration	Mucous layer	1 (20.0)	4 (80.0)	0.046
	Muscular layer	5 (71.4)	2 (28.6)	
	Full layer	57 (76.0)	18 (24.0)	
Lymph metastasis	Yes	44 (81.5)	10 (18.5)	0.012
	No	18 (56.2)	14 (43.8)	

Supplementary Table S2: Univariate analyses of various prognosis parameters in 86 CRC patients using cox regression model

	B	SE	Wald	P	95%CI
DcR3	-1.322	0.491	7.245	0.007	0.102±0.698
Age	0.037	0.014	7.085	0.008	1.010±1.066
Gender	-0.129	0.353	0.133	0.715	0.440±1.756
Differentiation	-0.041	0.190	0.045	0.831	0.661±1.394
Infiltration	1.478	0.721	4.203	0.040	1.067±18.013
Lymph metastasis	1.745	0.376	21.592	<0.001	2.743±11.957
